# Percutaneous Endoscopic Discectomy via Transforaminal Route for Discal Cyst

**DOI:** 10.1155/2015/273151

**Published:** 2015-08-19

**Authors:** Subash C. Jha, Kosaku Higashino, Toshinori Sakai, Yoichiro Takata, Mitsunobu Abe, Akihiro Nagamachi, Shoji Fukuta, Koichi Sairyo

**Affiliations:** Department of Orthopedics, Tokushima University, 3-18-15 Kuramoto, Tokushima 770-8503, Japan

## Abstract

*Background*. Discal cyst has been identified as a rare cause of low back pain and radiating leg pain. The pathogenesis and management of this condition are still debated. The largest number of reported cases had undergone microsurgery while very few cases have been treated with percutaneous endoscopic discectomy (PED). *Methods*. An 18-year-old boy complained of low back pain radiating to right leg after a minor road traffic accident. Diagnosis of a discal cyst at L4-L5 level was made based on magnetic resonance imaging (MRI). Despite conservative management for 6 months, the low back pain and radiating leg pain persisted so surgical treatment by PED was performed under local anesthesia. As the patient was a very active baseball player, his physician recommended a minimally invasive procedure to avoid damage to the back muscles. *Results*. The patient's low back pain and leg pain disappeared immediately after surgery and he made a rapid recovery. He resumed mild exercise and sports practice 4 weeks after surgery. Complete regression of the cystic lesion was demonstrated on the 2-month postoperative MRI. *Conclusion*. A minimal invasive procedure like PED can be an effective surgical treatment for discal cyst, especially in active individuals who play sports.

## 1. Introduction

Discal cysts are a rare cause of low back pain and radiculopathy, occurring in relatively young individuals [1–4]. Discal cysts are defined as intraspinal extradural cysts with distinct communication with the corresponding intervertebral disc [1, 3, 5]. The pathogenesis is still unclear.

The literature describes various treatments for discal cyst, from conservative to surgical excision [1, 4]. Percutaneous endoscopic discectomy (PED) is a minimally invasive technique especially for avoiding damage to the back muscles [5–7]. The prolapsed disc materials can be removed using the transforaminal approach under local anesthesia. It requires an 8 mm skin incision only. Traditional open surgery is the most commonly reported surgical treatment for discal cyst. Reports of use of PED for discal cyst in the literature are sparse. Herein, we report a case of successful treatment of discal cyst by PED under local anesthesia in a very active adolescent male baseball player.

## 2. Case Report 

### 2.1. History and Clinical Presentation

**Figure 1:**
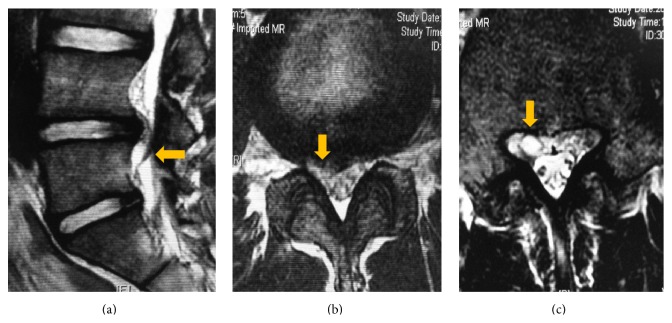
Preoperative magnetic resonance imaging: (a) sagittal T2-weighted image, (b) axial T2-weighted image through the L4-L5 disc, and (c) axial T2-weighted image through L5 pedicle demonstrate the extradural cystic lesion with high signal intensity, communicating with the corresponding intervertebral disc (arrows).

**Figure 2:**
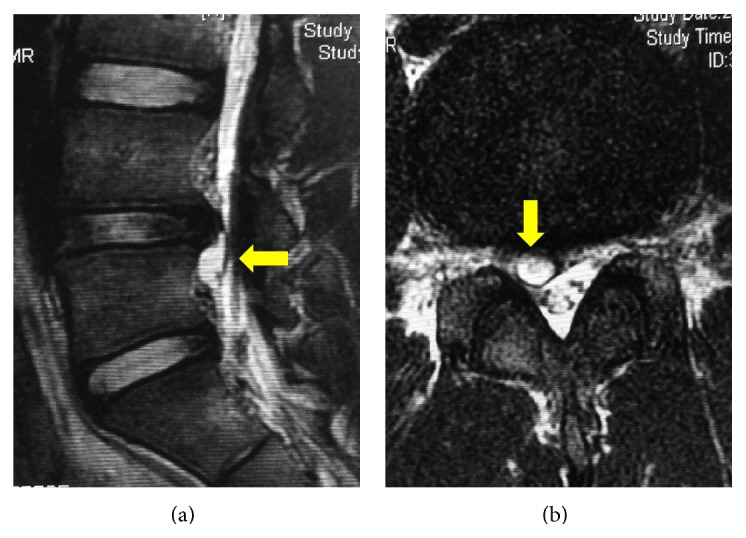
Preoperative magnetic resonance imaging at the 6-month follow-up: (a) sagittal T2-weighted and (b) axial T2-weighted images demonstrating the persistence of the cystic lesion (arrows).

An 18-year-old high school baseball player presented with low back pain radiating to the right lower limb after a minor road traffic accident. On physical examination, the straight leg raising test was positive at 45 degrees on the right side but the rest of his neurological examination was unremarkable. No abnormalities were seen on the plain radiograph. Magnetic resonance imaging (MRI) demonstrated an intraspinal extradural space-occupying lesion just caudal to the L4-L5 intervertebral disc on the right side. It was isointense on the T1-weighted image and hyperintense on T2-weighted sequence, suggesting the lesion was a discal cyst (Figure 1). He was managed conservatively as it was his first visit and his symptoms were relatively mild. He continued to play baseball while taking nonsteroidal anti-inflammatory drugs. His symptoms had persisted at the 3-month and 6-month follow-up consultations, and a repeat MRI showed the persisting discal cyst (Figure 2). The patient and his family were willing to undertake minimally invasive surgery for the discal cyst during the baseball off-season and they were referred to our department for PED. The patient was planning to return to baseball after the off-season prior to entering college.

### 2.2. Surgery

PED using the transforaminal technique was conducted under local anesthesia with the patient in the prone position on a standard spine frame. The optimal location for cannula insertion was determined preoperatively based on CT and MRI. The insertion point was selected 10 cm from the midline, though individual variation exists. Under C-arm guidance, local anesthesia was performed with 1% lidocaine. The 18G spinal needle was safely inserted into the intervertebral disc using the “walking technique” maneuver.

**Figure 3:**
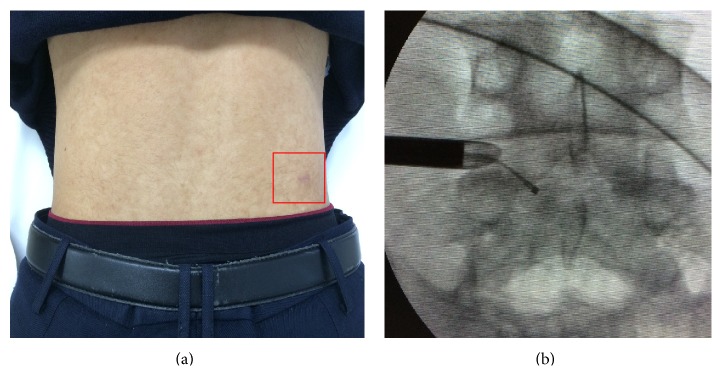
(a) Clinical appearance of the surgical scar after the procedure. (b) Confirmation of the residual cystic mass at the ventral epidural space by a probe.

Next, discography was conducted and indigo carmine was used to dye the nucleus pulposus blue, helping to identify the herniated fragment. A guide pin was inserted into the disc through the puncture needle, and the obturator and cannula were inserted sequentially through the 8-mm skin incision (Figure 3(a)). The patient's leg pain disappeared after insertion of the 8-mm cannula. This might have indicated shrinkage of the discal cyst after disruption of the cyst wall.

First, the disc fragment at the base of the herniated mass was removed. Using the inside-out and hand-down technique, the cannula was moved towards the epidural space, and extruded disc fragments were then removed. No residual cystic mass was observed on the ventral fluoroscopic view, and absence of a palpable mass was also confirmed by a probe (Figure 3(b)).

### 2.3. Postoperative Status on Follow-Up

**Figure 4:**
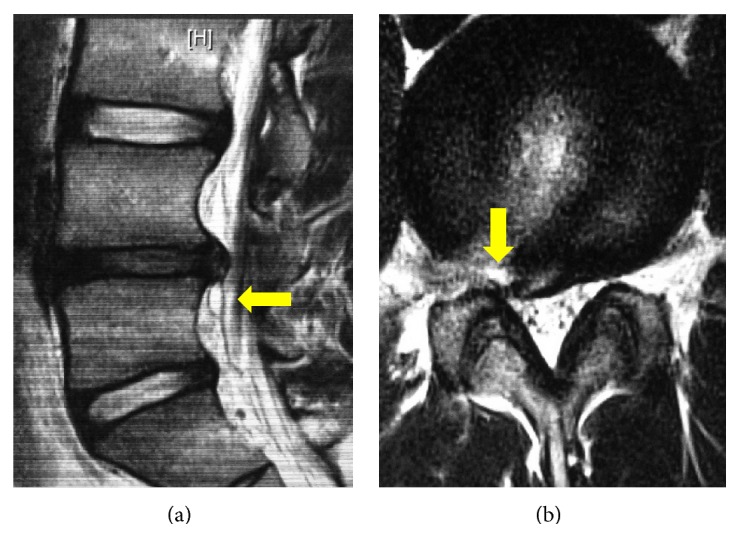
Postoperative magnetic resonance imaging at the 1-month follow-up: (a) sagittal T2-weighted and (b) axial T2-weighted images demonstrating the irregular posterior margin of the L4-L5 disc and decrease in size of the cyst (arrows).

**Figure 5:**
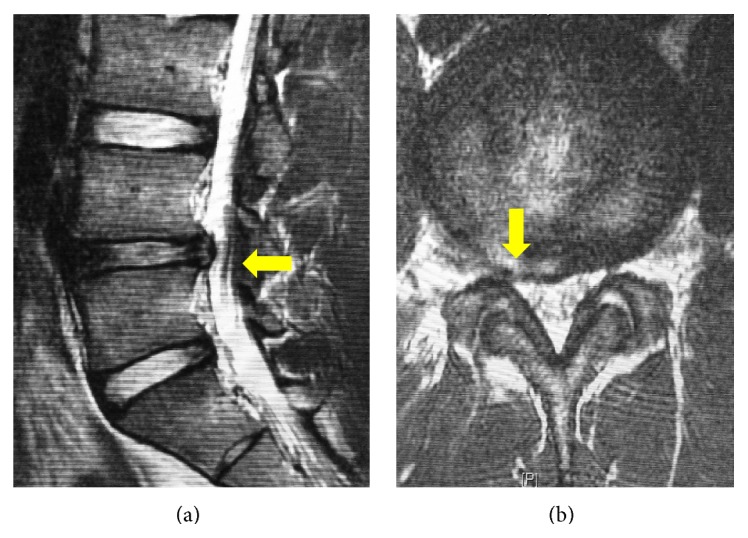
Postoperative magnetic resonance imaging at the 2-month follow-up: (a) sagittal T2-weighted and (b) axial T2-weighted images demonstrating complete resolution of the cystic lesion (arrows).

There was improvement in pain and straight leg raising test immediately after surgery. The visual analog scale for the leg pain decreased from 7/10 preoperatively to zero postoperatively. At the 1-month follow-up, MRI demonstrated a decrease in size of the discal cyst with an absence of residual symptoms (Figure 4). At the 2-month follow-up, MRI demonstrated complete resolution of the discal cyst (Figure 5). The patient had started stretching and trunk muscle isometric training just after surgery until 1 month afterwards. Between the first and second month after surgery, he did conditioning, jogging, and catch-ball and returned to competitive pitching 2 months after surgery.

## 3. Discussion

Clinically, it is almost impossible to distinguish discal cyst from other causes of low back pain with radiculopathy (e.g., HNP, perineural cyst, extradural arachnoid cyst, and cystic schwannoma) [1–4]. In general, most patients with discal cyst are relatively young and active and are predominantly male [1]. The pathogenesis of discal cyst remains unclear. In the epidural hematoma theory by Chiba et al., they postulated that discal cyst is the sequel of unabsorbed hemorrhage from the epidural venous plexus [8]. Kono et al. insisted that it was focal degeneration of the intervertebral disc causing an inflammatory response and formation of a reactive pseudomembrane that result in development of discal cyst [9]. The pathogenesis of discal cyst is more easily explained if it develops in the setting of acute and stressful mechanical impact [1]. Histopathological findings are predominantly fibrous connective tissue without synovial lining cells [1, 3]. Cyst content varies from serous to mucinous. Aydin et al. reported that hemorrhagic content in the cyst and/or hemosiderin deposits was present in 30.4% of cases [1].

Contrast flow into the cyst as demonstrated by discography is pathognomonic for discal cyst [1]. Lee et al. described the detailed MRI features of discal cyst, which makes MRI a very sensitive and primary diagnostic tool for its identification [1, 10]. MRI also better demonstrates the relation of the lesion with the surrounding structure. In the present case, we diagnosed the cyst based on MRI findings. Notably, the connection of the prolapsed disc and the cyst was obvious on MRI sagittal images, as shown in Figures 1 and 2.

A few cases of discal cyst that spontaneously regressed on conservative management have been reported [11, 12]. Most of the surgically treated cases were managed by partial hemilaminectomy and microscopic resection of the cyst under general anesthesia. The other minimally invasive option would be CT-guided aspiration [1]. Many surgeons believe that there is a high likelihood of recurrence after CT-guided aspiration, so they are more inclined to perform radical excision of the intraspinal cyst along with the corresponding communicating intervertebral disc [1].

The current case had prolapsed disc fragment of the discal cyst. To decompress the nerve root, the prolapsed disc should be removed, followed by removal or decompression of the discal cyst. To achieve this, we selected the transforaminal approach for PED. Transforaminal PED is a notable approach that fulfills the demands of discal cyst surgery [13, 14]. It can decompress the corresponding intervertebral disc and the adjacent discal cyst. Few reports of endoscopic transforaminal and interlaminar approaches for discal cyst resection with favorable results and no recurrence have been reported [5, 6]. We believe that complete excision of the cyst wall through the transforaminal approach is not practically possible, but decompression of cystic content and the corresponding intervertebral disc potentially decompresses the discal cyst, thereby relieving the symptoms. Additionally, the degenerated nucleus pulposus is stabilized by thermal annuloplasty using radiofrequency coagulation, thereby reducing the chances of recurrence [5, 14].

PED is less traumatizing and is minimally invasive, especially for the back muscles, and is performed under local anesthesia without resection of bone or ligament [5, 7]. The operating time is short, and postoperative rehabilitation is easy and fast. It is more advantageous cosmetically as the postoperative scar is around 8 mm. These advantages make it the more preferable approach for discal cysts compared with other conventional techniques [5, 7]. The current patient was a very active high school baseball player who wanted to return to baseball as soon as possible before entering college. He could return to the same competitive level 8 weeks after surgery and did not have recurrence.

## 4. Conclusion

A less invasive procedure like PED is a more appropriate approach for surgical management of discal cyst since it predominantly affects young and active individuals.
